# Content of the Saponin Protodioscin in *Brachiaria* spp. from the Eastern Plains of Colombia

**DOI:** 10.3390/toxins9070220

**Published:** 2017-07-13

**Authors:** Maria C. Lozano, Nhora M. Martinez, Gonzalo J. Diaz

**Affiliations:** 1 Departamento de Farmacia, Facultad de Ciencias, Universidad Nacional de Colombia, Bogotá, D.C., Colombia; 2 Departamento de Produccion Animal, Facultad de Medicina Veterinaria y de Zootecnia, Universidad Nacional de Colombia, Bogotá, D.C., Colombia; nmmartinezr@unal.edu.co; 3 Laboratorio de Toxicologia, Facultad de Medicina Veterinaria y de Zootecnia, Universidad Nacional de Colombia, Bogotá, D.C., Colombia; gjdiazg@unal.edu.co

**Keywords:** *Brachiaria* spp., protodioscin, saponin, Colombian eastern plains

## Abstract

Protodioscin is used as a marker of saponin content that could cause hepatotoxicity in ruminants. In *Brachiaria* spp. from two regions of the Colombian Eastern Plains (east mountain range of the Andean—“piedemonte” and Ariari River Valley) were determined this metabolite at 14 and 28 days post-cutting under different climatic conditions. No protodioscin was detected in *B. dictyoneura* or *B. humidicola*. In *B. brizantha*, *B. decumbens* and *B. ruziziensis* x *B. decumbens* x *B. brizantha* (hybrid), protodioscin content corresponded to an interaction between species, post-cutting time and season. Concentrations ≥1% (minimum toxic level) were recorded in *B. decumbens* and the hybrid, and to a lesser extent in *B. brizantha*. The concentration of protodioscin was higher at 28 days, when the pastures are suitable for consumption. *B. brizantha* accumulated the lowest saponin concentration, whereas the hybrid had the highest levels, particularly in the “piedemonte” and during drought (3.37%). Dry season favored the protodioscin concentration in *B. decumbens* (in river valley) and in the hybrid (in “piedemonte”). In the latter, there was a positive correlation with temperature and a negative with humidity, which are typical characteristics of dry periods. This is the first report of protodioscin content in the hybrid.

## 1. Introduction

Colombia has about 23,000,000 head of cattle, with more than 21% presence in the Orinoco region [1], also called Colombian Eastern Plains (lat: 50° 00‣ 00‣‣ N long: 70° 30‣ 00‣‣ W, a neotropical ecoregion that begins in the foothills of the eastern Andes and extends along the course of the Orinoco River), whose soils are acid and rich in aluminium [2]. In order to improve cattle productivity, *Brachiaria* spp., a grass with forage potential and widely distributed in tropical and subtropical regions, have been introduced from Africa [3]. The most common species include *B. decumbens*, *B. brizantha*, *B. dictyoneura*, *B. humidicola* and the hybrid *B. ruziziensis* x *B. decumbens* x *B. brizantha* [4].

Although *Brachiaria* spp. is frequently used to feed herbivorous animals, several species have produced intoxication by steroidal saponins leading to hepatobiliary injury and photosensitization [5]. Some factors increase animal susceptibility to intoxication with saponins, among them species (sheep), age (young animals) and no previous exposition to the pasture [6].

Protodioscin and dioscin are the main steroidal saponins present in *Brachiaria* [7,8,9]. Both of these glycosides are hydrolyzed in the rumen to produce diosgenin. Diosgenin is then saturated and epimerized in the rumen forming epismilagenin, which is further conjugated with glucuronic acid in the liver. Conjugated epismilagenin is highly lithogenic and can be deposited in canaliculi causing hepatic injury and obstruction to the biliary flow. Blockage of the biliary flow causes photoactive phylloerythrin from chlorophyll to enter the circulation, causing dermal lesions [10,11].

Some other saponins are present in *Brachiaria* spp. that could contribute to toxicosis in ruminants; however, for several years protodioscin has been used as a biomarker of exposure to the steroidal saponins from this pasture. Both exposure to animals and variations of content in the grasses have been investigated by quantifying protodioscin [6].

The hepatogenic photosensitization caused by *Brachiaria* spp. occurs only sporadically. It is known that saponin content in plants is variable [12]; climatic factors (rainfall, temperature and relative humidity) and plant age affect its levels in the grass. Frequently, it is reported that sprouting pastures have higher saponin concentrations than mature ones, however higher levels have been found in older *Brachiaria* [6].

Photosensitization in ruminants is a frequent condition in the Colombian Eastern plains, and it is usually attributed by farmers to the ingestion of plants [13]. It is unknown whether the *Brachiaria* spp. ingested by cattle in this region contains steroidal saponins that may be associated with photosensitization. Additionally, little is known about how environment influences saponin concentrations in the plants.

The objective of this study was to quantify the content of protodioscin in the main *Brachiaria* species present in the Colombian Eastern Plains and to investigate possible differences in concentration due to different plant species, time of regrowth of the grass, and place and time of collection.

## 2. Results

None of the *B. humidicola* and *B. dictyoneura* samples had detectable levels of protodioscin. Therefore, only the results for the other three species (*B. brizantha*, *B. decumbens* and the hybrid *B. ruziziensis* x *B. decumbens* x *B. brizantha*) are presented.

The highest average content of protodioscin was found in *B. decumbens* (1.98% ± 0.68%, with values ranging from 0.59% to 2.99%), followed by the hybrid (1.74% ± 0.67%, 0.77% to 3.68%) and *B. brizantha* (1.09% ± 0.36%, 0.47% to 1.81%). A total of 77% of the samples contained protodioscin concentrations above 1%, and corresponded to 30% for hybrid, 27% for *B. decumbens* and 20% for *B. brizantha*. Only seven samples (5% of the total) contained protodioscin values ≥3%, five corresponded to the hybrid from “piedemonte” and two were *B. decumbens* from the alluvial valley (Table 1).

**Figure 1:**
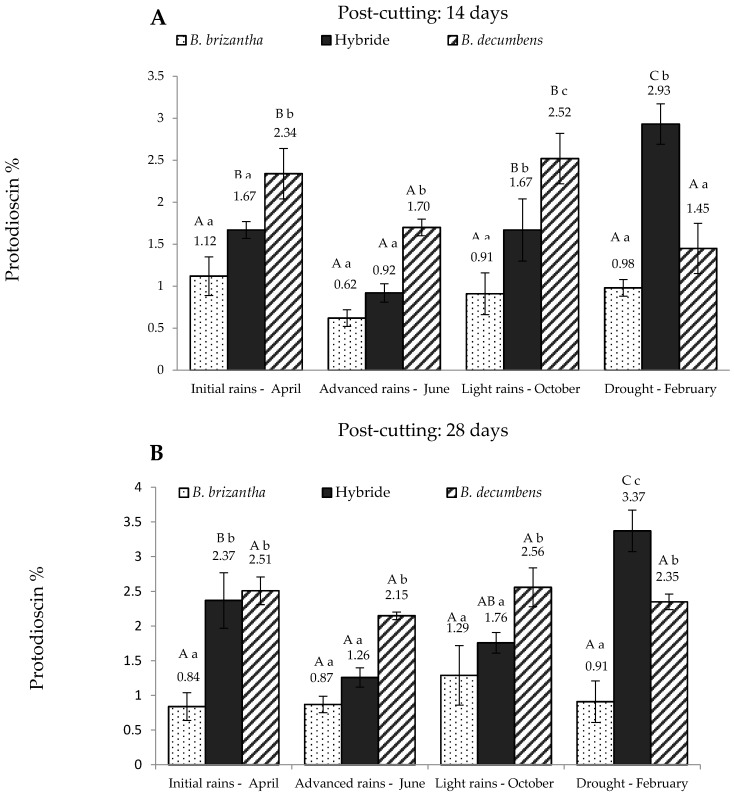
Protodioscin concentration in *Brachiaria* spp. (based on dry matter) from “piedemonte” in Colombian Eastern Plains; at 14 days (**A**) and 28 days (**B**) post-cutting. Hybrid: *B. ruziziensis x B. decumbens x B. brizantha. Lowercase letters indicate statistical differences between species of the same post-cutting time at the same season. Capital letters indicate statistical differences in the same species of the same post-cutting time between seasons. n = 3. *p* < 0.05.*

**Figure 2:**
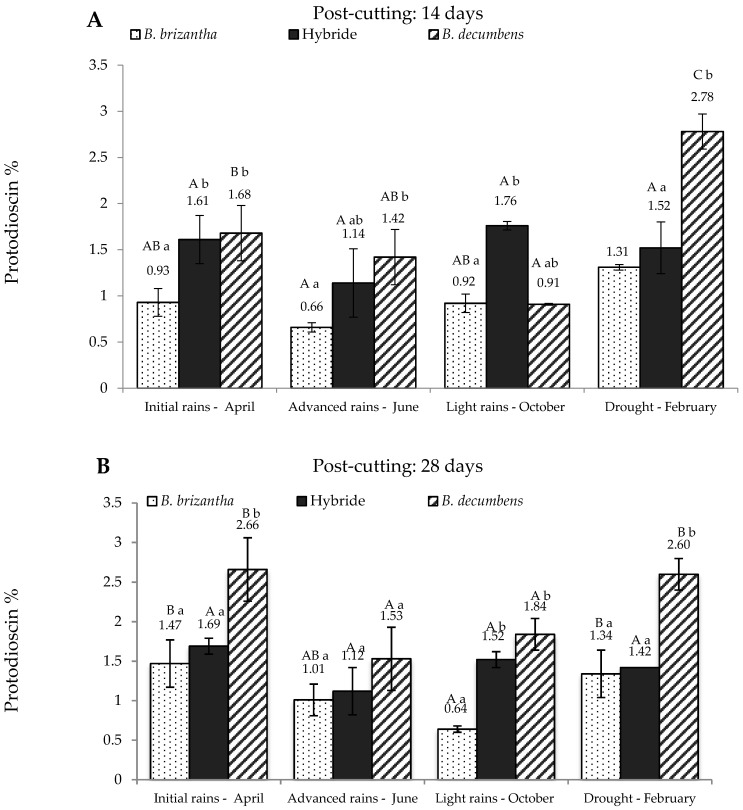
Protodioscin concentration in *Brachiaria* spp. (based on dry matter) from Ariari river valley in Colombian Eastern Plains; at 14 days (**A**) and 28 days (**B**) post-cutting. Hybrid: *B. ruziziensis x B. decumbens x B. brizantha. Lowercase letters indicate statistical differences between species of the same post-cutting time at the same season. Capital letters indicate statistical differences in the same species of the same post-cutting time between seasons. n = 3. *p* < 0.05.*

According to the factorial model, there was an interaction between the *Brachiaria* spp., season and post-cutting time (*p* < 0.05), suggesting that protodioscin concentration depends on these three factors. Figure 1 shows the values obtained for these three variables for the “piedemonte” region and Figure 2 for the alluvial valley of the Ariari river. In regard to the post-cutting time, it was found that the hybrid had significantly higher protodioscin levels (*p* < 0.05) at 28 days in all periods except October. A similar trend was observed for *B. decumbens* in advanced rains (June) and drought (*p* < 0.05). At 14 days post-cutting (Figure 1A), *B. brizantha* had the lowest levels of protodioscin at all sampling times, whereas *B. decumbens* had significantly higher levels on the first three sampling times. The hybrid showed intermediate levels of saponin in the first three sampling times.

Its protodioscin content was lowest in June and then increased linearly in the two following sampling times reaching its maximum level in the dry season. At this point, protodioscin content in the hybrid was about two and three times higher than that of *B. decumbens* and *B. brizantha*, respectively. Protodioscin levels in “piedemonte” at 28 days post-cut (Figure 1B) showed a similar pattern: *B. brizantha* had the lowest levels of protodioscin; *B. decumbens* had the highest levels of protodioscin in the first three sampling periods, and the hybrid had the lowest protodioscin levels in the “advanced rains” period.

Figure 2 shows protodioscin content in *Brachiaria* spp. from the alluvial valley. At 14 days after re-growth (Figure 2A), *B. brizantha* produced the lesser amounts of protodioscin, whereas *B. decumbens* produced the highest, except for the “light rains” season. Once again, the hybrid showed a tendency to concentrate intermediate levels of the saponin and these levels do not show significant differences at any sampling time. Figure 2B shows the results at 28 days post-cutting. Again, *B. brizantha* showed the lowest concentration of saponin, while *B. decumbens* has the highest concentration, doubling and tripling the levels present in *B. brizantha* in drought and light rains respectively. The hybrid showed similar results compared to 14 days post-cutting.

**Figure 3:**
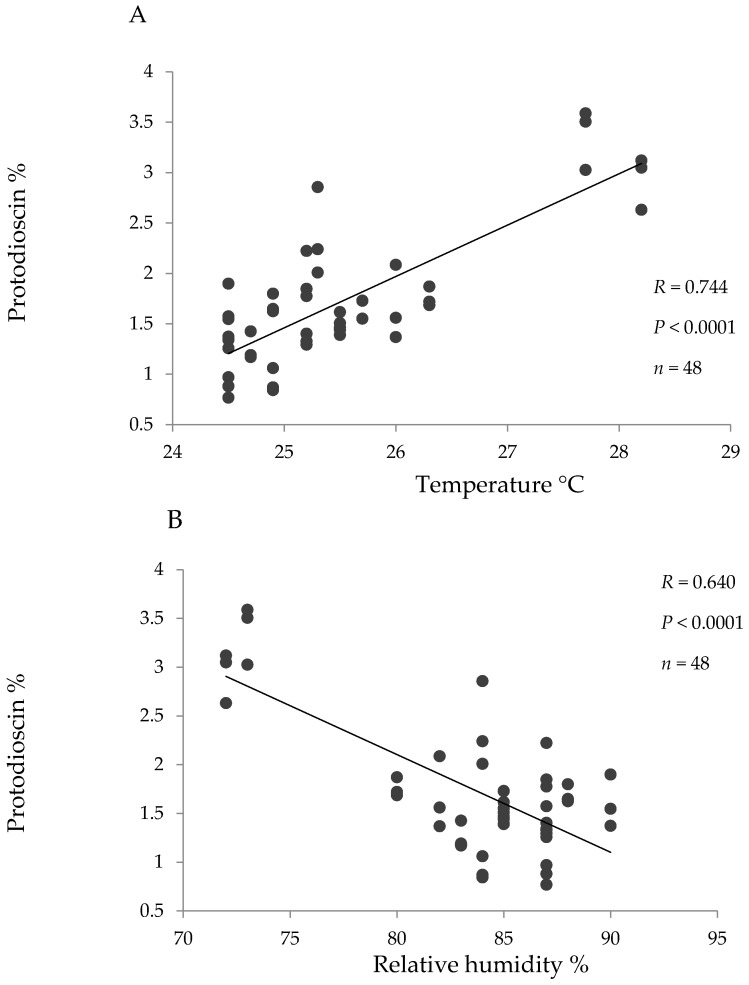
Linear correlation between the environmental parameters temperature (**A**) and relative humidity (**B**) and protodioscin concentration (based on dry matter) for the hybrid (*B. ruzizienzis* x *B. decumbens* x *B. brizantha*).

Pearson correlation coefficients were high and significant between temperature and protodioscin content and between relative humidity and protodioscin content for the hybrid (Figure 3). No significant correlations were seen between protodioscin levels and precipitation or solar brightness, or between saponin level and climatic factors in *B. decumbens* and *B. brizantha*.

## 3. Discussion

From the three species found to contain detectable levels of protodioscin in the present study, *B. brizantha* and *B. decumbens* have been traditionally related to toxicosis in ruminants, whereas this is the first time that the hybrid *B. ruziziensis* x *B. decumbens* x *B. brizantha* has been analyzed for protodioscin. It has been reported that *B. decumbens* contains higher protodioscin levels than *B. brizantha* [6] and the results found in the present trial show that the hybrid can contain even higher levels than *B. brizantha* and *B. decumbens*, making this pasture a potential threat for secondary photosensitization. In fact, in a recent case involving goats in Cuba, a high mortality (16%) and morbidity (50%) was associated with acute hepatic cholestasis attributed to the intake of hybrid grass; however, no attempts to determine its saponin content were made [14].

In regard to post-cutting time, there were large differences in protodioscin content between 14 and 28 days. Depending on the time and place of sampling, protodioscin content increased by about 15% to 62% at 28 days of regrowth compared to 14 days. In previous studies, the effect of pasture age on *Brachiaria* spp. saponins had shown variable results. Increased protodioscin levels as the age and reproductive status of *B. decumbens* and *B. brizantha* increased was reported by Brum et al. (2009) [15], while others found the highest concentrations in younger pastures [16,17]. In the present study, it was found that there is a higher protodioscin content at 28 days post-cutting, when *Brachiaria* spp. is in the rapid growth phase and accumulates high nutritive value dry matter [18], suggesting that the synthesis of saponins is favored by a positive energy balance in the grass.

The minimum protodioscin concentration causing toxicosis in sheep (the most sensitive species) appears to be 1% [6]. Many of the *B. decumbens* and hybrid samples contained levels ≥1%, while *B. brizantha* samples exceeded this threshold value mainly in the alluvial valley of the Ariari river at 28 days post-cutting time. These results suggest that pastures of *B. decumbens* and the hybrid in the Colombian Eastern Plains could cause intoxication at different times of the year, whereas *B. brizantha* would represent a risk mainly in the stream of the Ariari river at 28 days of regrowth. It is important to consider that other saponins with diosgenin, whose metabolites induce hepatic lesions, have been identified in *B. decumbens* and *B. brizantha* [19,20,21]. So, a better biomarker for predicting toxicosis could be considered in future studies, including determination of other saponins (dichotomin, saponine B, dioscin) or diosgenin quantification, as is suggested by Low, 2015 [22].

It is expected that protodioscin levels vary throughout the year and in the present study protodioscin levels were particularly higher during drought (for *B. brizantha* and *B. decumbens* in the alluvial valley and for the hybrid in “piedemonte”). Further, in the hybrid grass a high positive correlation was found between protodioscin content and temperature, and a high negative correlation between protodioscin and humidity. Both a rise in temperature and a decrease in humidity are characteristic during drought. If steroidal saponins are used by the plant as a defense mechanism [23] and water deficit makes it more susceptible to insect attack [24], the higher levels of protodioscin found in drought season could represent a defense response of these pastures.

Large differences in saponin content depending on the geographical location of the crops have been reported. For instance, while *Tribulus terrestris* from southeast Europe has protodioscin concentrations up to 1027 μg/g, the same plant species collected in Western Asia has maximum levels of 32 μg/g [25]. In the present study, there was a tendency for higher protodioscin levels in the “piedemonte”. Differences in the content of plant saponins and in particular those dependent on geographical origin are difficult to interpret. The characteristics of each crop site vary, including soil fertility [26]. Soils from the Orinoco region are characterized by acidic pH and high aluminium content that makes them unproductive [27]. Some studies have shown that saponin synthesis is enhanced under stress conditions suggesting that these compounds may be involved in adapting the plant to survival under adverse soil circumstances [23]. On the other hand, secondary metabolites can be high in low fertility soils [28]. It is possible that the typical characteristics of the Colombian Eastern Plains soils (acid pH and high aluminium content) are responsible for the high protodioscin concentrations found in the present study.

In summary, the results of the present study show that 77% of the samples tested contain ≥1% protodioscin, a cut-off limit for potential toxicosis. The highest protodioscin concentrations were generally reached 28 days after the beginning of the re-growth, a time of positive energy balance for the plant, and the moment when the animals eat it. Assuming that protodioscin content can be used as a marker metabolite for potential toxicosis (as it has been done in multiple investigations), the consumption of *B. decumbens* (in alluvial valley) and the hybrid grass (in “piedemonte”), should be avoided, particularly in the dry season. However, it is important to consider other saponins present in *Brachiaria* spp., or even diosgenin for better prediction of saponin content in grass.

Since making hay reduces protodioscin content [29], using the grasses in the form of hay could be an alternative in order to avoid saponin toxicosis. Also, it would be interesting to consider the use of other grasses for cattle growing, particularly native grasses previously tested to verify that they do not accumulate saponins or other potentially toxic compounds.

## 4. Materials and Methods

### 4.1. Region of Study

In the Colombian Orinoco region, the Department of Meta has the greatest development in livestock production, which has led to the introduction of better pastures. Introduction of exotic grasses has been particularly important in the plains located closer to the east mountain range of the Andean region (known locally as “piedemonte”) and in the alluvial valleys of this Department [30]. Climate in this region is characterized by a rainfall of over 3000 mm/year, comprising a rainy season of eight months (April to November) and a drought of four (December to March). The average temperature is 26 °C and the relative humidity ranges from 60 to >80% [31].

Two farms with established pastures of the main *Brachiaria* species (*B. brizantha*, *B. decumbens*, *B. dictyoneura*, *B. humidicola*, and the hybrid *B. ruziziensis* x *B. decumbens* x *B. brizantha*) were selected, one of them located in “piedemonte” (Villavicencio, Apiay—lat: 4.0°3.0′ N long: 73.0° 28.0′ W) and the other in the alluvial valley of the Ariari river (San Martín de los Llanos, La Reforma—lat: 3.0° 3.7′ long: 73.0° 44.0). Photosensitization cases had been previously reported in these two farms.

### 4.2. Sampling

Established pastures of the five species of *Brachiaria* spp. were sampled, according to the rotation scheme of the area, which consists of introducing cattle to pastures that have been free of animals for a period of approximately 28 days. Plant material was collected at 14 (rapid growth phase) and at 28 days (slow growth phase) after the rest period had started. Three representative points in each paddock with an area of 1 m^2^ were selected, cutting the grass at the height that animals usually do (2/3 distal). The same points were sampled at four different seasons, corresponding to initial rains (April 2012, characterized by a previous month of precipitation), advanced rains (June 2012, characterized by four previous months of precipitation), light rains (October 2012, with lower precipitation, after a semi-dry period) and droughty (February 2013, with low precipitation). The climate information (temperature, relative humidity, precipitation and solar brightness) for each point and sampling period was obtained from meteorological stations of the “Instituto de Hidrología, Meteorología y Estudios Ambientales—IDEAM”.

**Figure 4:**
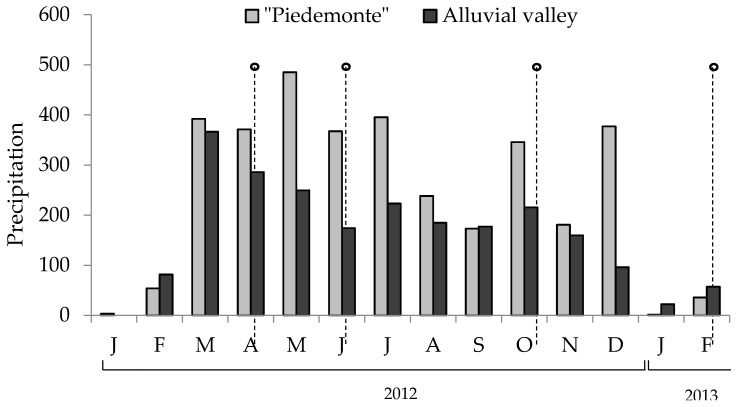
Precipitation in “piedemonte” and Ariari river valley during the sampling period. Points indicate the moment when the pastures were collected.

Figure 4 shows rain precipitation at the time the pastures were sampled. Table 2 shows the climate parameters for each of the two harvesting times (14 and 28 post-cut days) in the four seasons sampled. All samples were dried at 60 °C in a forced-ventilation oven for 48 hours, ground in a knife mill and analyzed for protodioscin levels as described below.

### 4.3. Determination and Quantification of Protodioscin

For the extraction of protodioscin the protocol from [32] was used, with slight modifications, particularly related to solvent volume (twice the amount was used) and ultrasonication time (the same extraction time for all extractions was used). To one gram of the dried and milled plant material, 15 mL of 50:50 acetonitrile: H_2_O were added. The sample was then extracted by ultrasonication for 30 min and then centrifuged (700 g × 5 min) in order to recover the supernatant. This procedure was repeated two more times adding 10 mL of the acetonitrile: H_2_O to the pellet at each time. The extracts obtained were pooled and brought to a volume of 50 mL using a volumetric flask. A fraction of the extract was filtered through a 0.45 μm PTFE (polytetrafluorethylene) membrane and diluted 1:4 with 50:50 acetonitrile: H_2_O.

For the determination and quantification of protodioscin the high-performance liquid chromatographic (HPLC) method described by [33] was used, without modifications.

**Figure 5:**
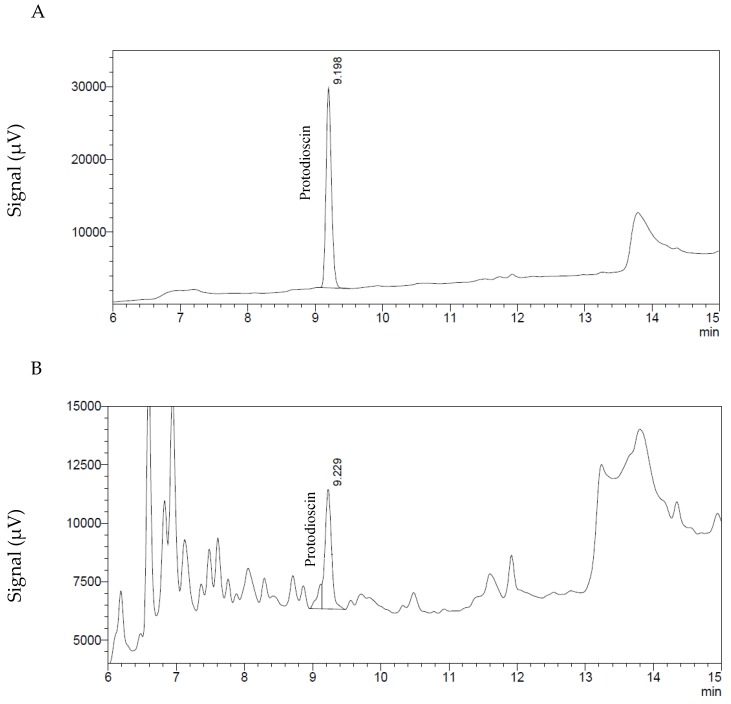
Chromatograms of protodioscin. (**A**) Standard solution of 500 μg/mL. (**B**) Extract of a *B. decumbens* sample containing protodioscin at a concentration equivalent to 2.22% in the dried grass sample.

The HPLC system (Shimadzu Scientific Instruments—Columbia, MD, USA) consisted of a DGU-20A3 degassing unit, an LC-20AB pump, a SIL-20A HT autosampler, a CTO-20A column oven and a UV-vis SPD-20AV, all controlled by the software “LC Solutions”. The separation was performed on a Alltima Spherisorb ODS-2 (5 μm, 150 × 4.6 mm, Grace Alltech—Columbia, MD, USA) column protected with a precolumn of the same stationary phase (7.5 × 4.6 mm ODS-2, 5 μm). The mobile phase was acetonitrile: H_2_O (water acidified with 0.5% H_3_PO_4_), in a linear gradient from 5% to 100% acetonitrile from 0 to 20 min and remaining at 100% acetonitrile for 5 min. From 25 to 30 min, the mobile phase was again 5% acetonitrile and 95% water. The flow rate was 1 mL/min and the protodioscin was monitored at 210 nm. Two μL of the sample were injected into the HPLC system. Under these conditions the retention time of the protodioscin standard (ChromaDex—Irvine, CA, USA) was 9.2 min (Figure 5). The linearity was established with concentrations of 10, 25, 100, 200, 400, 500, 600, 800 and 1000 μg/mL, a CV (coefficient of variation) of 5.1% was determined for the response factors. Limit of detection was 2.8 μg/mL and limit of quantification limit 8.3 μg/mL, equivalent to a sample concentration of 0.05% and 0.16%, respectively. These values were obtained according to EPA (Environmental Protection agency) 40 CFR (Code of Federal Regulations) Part 136, Appendix B [34].

To determine which factors (plant species, season, time of re-growth) were most important in determining protodioscin concentration, a completely randomized 5 x 4 x 2 factorial design model was used. This model corresponds to five brachiaria species (*B. brizantha*, *B. decumbens*, *B. dictyoneura*, *B. humidicola* and the hybrid—*B. ruziziensis* x *B. decumbens* x *B. brizantha*), four seasons (initial rains, advanced rains, light rains and droughty) and two post-cutting times (14 and 28 days). The model was applied to the results obtained in “piedemonte” and to the alluvial valley of the Ariari river. The interest was focused on finding possible differences between species in the same season, between seasons for the same species and between post-cutting time in the same species and season. This model fulfilled the assumption of homogeneity of variances according to the O’Brien test [35]. Also, in order to identify the possible effect of climatic factors on protodioscin content, a Pearson’s correlation was calculated between the environmental parameters (temperature, precipitation, relative humidity and solar brightness) and protodioscin concentration. This analysis was applied for all data, as well as for each of the regions, post-cutting time and *Brachiaria* species analyzed. The SAS 9.2 software was used to perform the statistical analysis, accepting a significance of *p* < 0.05.

## Figures and Tables

**Table 1:** Percentage of *Brachiaria* spp. sampling in “piedemonte” (P) and Ariari river valley (V) in the -Department of Meta with different protodioscin levels.

Protodioscin Concentration (%)	*Brachiaria decumbens*		Hybrid *		*Brachiaria brizantha*		Total
	V	P	V	P	V	P	
>0 to ≤1	0% *n* = 0	4% *n* = 6	1% *n* = 2	1% *n* = 2	8% *n* = 11	4% *n* = 5	23% *n* = 33
>1 to ≤2	4% *n* = 6	5% *n* = 7	8% *n* = 12	14% *n* = 20	8% *n* = 12	12% *n* = 17	51% *n* = 74
>2 to ≤3	11% *n* = 16	6% *n* = 8	3% *n* = 5	1% *n* = 1	0% *n* = 0	0% *n* = 0	21% *n* = 30
>3	0% *n* = 0	1% *n* = 2	4% *n* = 5	0% *n* = 0	0% *n* = 0	0% *n* = 0	5% *n* = 7

* B. ruziziensis x B. decumbens x B. brizantha.

**Table 2:** Climate parameters during *Brachiaria* spp. sampling in “piedemonte” (P) and Ariari river valley (V) in the Department of Meta.

Sampling	Day	*P* (mm)		*T* (°C)		RH(%)		SB(hours)	
		P	V	P	V	P	V	P	V
April 2012 Initial rains	14	130.4	183.8	25.7	24.5	85	90	76.0	37.1
	28	367.0	276.9	25.3	24.9	84	88	133.5	91.8
June 2012 Advanced rains	14	176.0	112.7	24.9	24.5	84	87	55.5	58.7
	28	364.9	203.5	24.7	24.5	83	87	136.4	128.5
October 2012 Light rains	14	129.5	159.0	26.0	25.2	82	87	51.3	60.2
	28	286.6	244.8	26.3	25.5	80	85	159.1	131.6
February 2013 Droughty	14	9.4	23.9	28.2	25.2	72	87	70.8	74.8
	28	35.6	57.2	27.7	25.5	73	85	116.8	122.9

P: precipitation, T: temperature, RH: relative humidity, SB: solar brightness. Values of temperature and relative humidity represent the average from day zero to the day of sampling, while the other parameters represent the sum in the same period. Source: IDEAM. La Libertad – Villavicencio Meta metereological station. Barbascal—San Martín de los Llanos Meta metereological station.

## References

[1] ICA (Instituto Colombiano Agropecuario) Censo Pecuario Nacional 2016. http://www.ica.gov.co/getdoc/8232c0e5-be97-42bd-b07b-9cdbfb07fcac/Censos-2008.aspxCensoPecuarioNacional-2016.

[2] WWF (World Wild Foundation) Tropical and Subtropical Grasslands, Savannas and Shrublands. Nothern South America in Colombia and Venezuela. https://www.worldwildlife.org/ecoregions/nt0709.

[3] Argell P., Keller-Grein G., Miles B.L., Maass C.B. (1996). Regional experience with *Brachiaria*: Tropical America-Humid Lowlands. Brachiaria: Biology, Agronomy and Improvement.

[4] Lozano M., Doncel B., Moreno C. (2011). Manual de Plantas Tóxicas Para Bovinos. Región Llanos Orientales de Colombia: Meta y Casanare.

[5] Diaz G. (2010). Plantas Tóxicas de Importancia en Salud y Producción Animal en Colombia.

[6] Riet-Correa B., Castro M.B., De Lemos R.A., Riet-Correa G., Mustafa V., Riet-Correa F., Lemos R. (2011). *Brachiaria* spp. poisoning of ruminants in Brazil. Pesq. Vet. Bras..

[7] Brum K., Haraguchi M., Lemos R., Riet-Correa F., Fioravanti M. (2000). Crystal-associated cholangiopathy in sheep grazing *Brachiaria decumbens* containing the saponin protodioscin protodioscin. Pesq. Vet. Bras..

[8] Cruz C., Driemeier D., Pires V., Colodel E., Daketa A. (2000). Isolation of steroidal sapogenins implicated in experimentally induced cholangiopathy of sheep grazing *Brachiaria decumbens* in Brazil. Vet. Hum. Toxicol..

[9] Cruz C., Driemeier D., Pires V., Schenkel E. (2001). Experimentally induced cholangiohepatopathy by dosing sheep with fractionated extracts from *Brachiaria decumbens*. J. Vet. Diagn. Investig..

[10] Miles C., Wilkins A., Munday S., Flayoen A., Holland P., Smith B. (1993). Identification of insoluble salts of the β-D-glucuronides of episarsasapogenin and epismilagenin in the bile of lambs with alved and examination of *Narthecium ossifragum*, *Tribulus terrestris* and *Panicum miliaceum* for sapogenins. J. Agric. Food Chem..

[11] Miles C., Wilkins A., Munday S., Holland P., Smith B., Lancaster M. (1992). Identification of the calcium salt of epismilagenin beta-D-glucuronide in the bile crystals of sheep affected by *Panicum dichotomiflorum* and *Panicum schinzii* toxicoses. J. Agric. Food Chem..

[12] Mysterud I., Flåøyen A., Loader J.I., Wilkins A.L. (2007). Sapogenin levels in *Narthecium ossifragum* plants and *Ovis aries* lamb faeces during two alveld outbreaks in Møre og Romsdal, Norway, 2001. Vet. Res. Commun..

[13] Torres P., Diaz G.J., Cárdenas E., Lozano M.C. (2012). Ethnobotanical Study of Plants Poisonous to Cattle in Eastern Colombia. IJPPR.

[14] Chong Dubé D., Figueredo J.M., Percedo M.I., Domínguez P., Martínez García Y., Alfonso P., Marrero Faz E. (2016). Toxicosis por pasto Mulato (*Brachiaria ruziziensis- Brachiaria brizantha*) en cabras de la provincia Artemisa. Rev. Salud Anim..

[15] Brum K., Haraguchi M., Garutti M., Nóbrega F., Rosa B., Fioravanti M., Soares J. (2009). Steroidal saponin concentrations in *Brachiaria decumbens* and *B. brizantha* at different developmental stages. Cienc. Rural.

[16] Castro M.B., Santos H.L., Mustafa V.S., Gracindo C.V., Moscardini A.C.R., Louvandini H., Paludo G.R., Borges J.R.J., Haraguchi M., Ferreira M.B., Riet-Correa F., Pfister J., Schild A.L., Wierenga T. (2011). *Brachiaria* spp. poisoning in sheep in Brazil: Experimental and epidemiological findings. Poisoning by Plants, Mycotoxins, and Related Toxin, Proceedings of the 8th International Symposium on Poisonous Plants, João Pessoa, Para-Íba, Brazil, 2–8 May 2009.

[17] Ferreira M., Brum K., Fernandes C., Martins C., Pinto G., Castro V., Rezende K.G., Riet-Correa F., Haraguchi M., Wysocki H.L., Riet-Correa F., Pfister J., Schild A.L., Wierenga T. (2011). Variation in saponin concentration in *Brachiaria brizantha* leaves as a function of maturation: Preliminary data. Poisoning by Plants, Mycotoxins, and Related Toxin, Pcoceedings of the 8th International Symposium on Poisonous Plants, João Pessoa, Para-Íba, Brazil, 2–8 May 2009.

[18] Sierra J. (2005). Fundamentos Para el Establecimiento de Pasturas y Cultivos Forrajeros.

[19] Lee S., Mitchell R., Gardner D., Tokarnia C., Riet-Correa F., Riet-Correa F., Pfister J., Schild A.L., Wierenga T. (2011). Measurement of steroidal saponins in *Panicum* and *Brachiaria* grasses in the USA and Brazil. Poisoning by Plants, Mycotoxins, and Related Toxin, Proceedings of the 8th International Symposium on Poisonous Plants, João Pessoa, Para-Íba, Brazil, 2–8 May 2009.

[20] Meagher L., Wilkins A., Miles C., Fagliari J. (1996). Hepatogenous photosensitization of ruminants by *Brachiaria decumbens* and *Panicum dichotomiflorum* in the absence of sporidesmin: Lithogenic saponins may be responsible. Vet. Hum. Toxicol..

[21] Pires V.S., Taketa A.T.C., Gosmann G., Schenkel E.P. (2002). Saponins and sapogenins from *Brachiaria decumbens* Stapf. J. Braz. Chem. Soc..

[22] Low S. (2015). Signal Grass (*Brachiaria decumbens*) Toxicity in Grazing Ruminants. Agriculture.

[23] Szakiel A., Pa̧czkowski C., Henry M. (2011). Influence of environmental biotic factors on the content of saponins in plants. Phytochem. Rev..

[24] Giraldo C., Reyes L., Molina J. (2011). Manejo Integrado de Artrópodos y Parásitos en Sistemas Silvopastoriles Intensivos.

[25] Dinchev D., Janda B., Evstatieva L., Oleszek W., Aslani M.R., Kostova I. (2008). Distribution of steroidal saponins in *Tribulus terrestris* from different geographical regions. Phytochemistry.

[26] Pavarini D.P., Pavarini S.P., Niehues M., Lopes N.P. (2012). Exogenous influences on plant secondary metabolite levels. Anim. Feed Sci. Technol..

[27] Viloria de la Hoz J. (2009). Geografía Económica de la Orinoquia.

[28] Gobbo-Neto L., Lopes N.P. (2007). Plantas medicinais: Fatores de influência no conteúdo de metabólitos secundários fatores que influenciam o conteúdo de metabólitos secundários. Quim. Nova.

[29] Lima F.G., Lee S.T., Pfister J.A., Miyagi E.S., Costa G.L., Dias R., Fioravanti M.C. (2015). The effect of ensiling and haymaking on the concentrations of steroidal saponin in two *Brachiaria* grass species. Cienc. Rural.

[30] Pulido J.I., Romero M., Rivero S.T., Duarte O.A., Gómez P.J., Vanegas E., Jaime W.E., Parra J.L., Pérez R.A., Cipagauta M. (2002). Atlas de los Sistemas de Producción Bovina. Modulo Orinoquía y Amazonía. Plan de la Modernización Tecnológica de la Ganadería Bovina Colombiana.

[31] Rippstein G., Escobar G., Motta F. (2001). Agroecología y Biodiversidad de las Sabanas en los Llanos Orientales de Colombia. Meta.

[32] Lima F.G., Haraguchi M., Pfister J.A., Guimaraes V.Y., Diogo D.F. (2012). Weather and plant age affect the levels of steroidal saponin and *Pithomyces chartarum* spores in *Brachiaria* grass main forage source for ruminants. IJPPR.

[33] Lee E.J., Yoo K.S., Patil B.S. (2010). Development of a Rapid HPLC-UV Method for Simultaneous Quantification of Protodioscin and Rutin in White and Green Asparagus Spears. J. Food Sci..

[34] EPA (Environmental Protection Agency) (2011). 40 CFR Appendix B to Part 136—Definition and Procedure for the Determination of the Method Detection Limit-Revision 1.11.

[35] Martínez R., Martínez N., Martínez M.V. (2011). Diseño de Experimentos en Ciencias Agropecuarias y Biológicas con SAS, SPSS, R Y STATISTIX.

